# Modulation of digestive physiology and biochemistry in *Mytilus californianus* in response to feeding level acclimation and microhabitat

**DOI:** 10.1242/bio.019430

**Published:** 2016-07-11

**Authors:** Kwasi M. Connor, Aaron Sung, Nathan S. Garcia, Andrew Y. Gracey, Donovan P. German

**Affiliations:** 1 Department of Ecology and Evolutionary Biology, University of California, Irvine, CA 92697, USA; 2 Department of Earth System Science, University of California, Irvine, CA 92697, USA; 3 Department of Biological Sciences, University of Southern California, Los Angeles, CA 90089, USA

**Keywords:** Clearance rate, Digestive enzyme activity, Growth, Rate-maximization, Respiration rate, Thermal stress, Yield-maximization

## Abstract

The intertidal mussel *Mytilus californianus* is a critical foundation species that is exposed to fluctuations in the environment along tidal- and wave-exposure gradients. We investigated feeding and digestion in mussels under laboratory conditions and across environmental gradients in the field. We assessed whether mussels adopt a rate-maximization (higher ingestion and lower assimilation) or a yield-maximization acquisition (lower ingestion and higher assimilation) strategy under laboratory conditions by measuring feeding physiology and digestive enzyme activities. We used digestive enzyme activity to define resource acquisition strategies in laboratory studies, then measured digestive enzyme activities in three microhabitats at the extreme ends of the tidal- and wave-exposure gradients within a stretch of shore (<20 m) projected sea-ward. Our laboratory results indicated that mussels benefit from a high assimilation efficiency when food concentration is low and have a low assimilation efficiency when food concentration is high. Additionally, enzyme activities of carbohydrases amylase, laminarinase and cellulase were elevated when food concentration was high. The protease trypsin, however, did not increase with increasing food concentration. In field conditions, low-shore mussels surprisingly did not have high enzyme activities. Rather, high-shore mussels exhibited higher cellulase activities than low-shore mussels. Similarly, trypsin activity in the high-shore-wave-sheltered microhabitat was higher than that in high-shore-wave-exposed. As expected, mussels experienced increasing thermal stress as a function of reduced submergence from low to high shore and shelter from wave-splash. Our findings suggest that mussels compensate for limited feeding opportunities and thermal stress by modulating digestive enzyme activities.

## Abstract (precis)

**Summary:** We show for the first time that the mussel *Mytilus californianus* has a flexible digestive strategy to deal with varying environmental conditions that are based upon their position on the shore.

## INTRODUCTION

The intertidal mussel *Mytilus californianus* aggregates to form dense beds along the western shores of North America. As a foundation species, *M. californianus* beds harbor up to ∼300 associated taxa, filter particulate organic matter from the water column, and serve as prey for an assortment of marine organisms; therefore, they provide important ecological services to shoreline communities (Suchanek, 1980). Because mussels are sessile ectotherms, they must cope with fluctuations in biological and physical factors of the prevailing intertidal environment, including tidal height, wave force, temperature, and food concentration, all of which can vary over small spatial scales (meters to tens of meters) (Connor and Robles, 2015; Dowd et al., 2013; Logan et al., 2012; Petes et al., 2007). Thus, understanding environmental-physiological dynamics over small scales can serve as a basis for comprehending how mussel population demographics and ranges may shift over geographic scales (hundreds of kilometers) (Helmuth, 2009).

Patterns of distribution and abundance in *M. californianus* are, in part, shaped by environmentally sensitive somatic growth rates and final body sizes, which vary greatly within a given expanse of rocky shoreline along horizontal transects (<20 m) (Connor and Robles, 2015). Size is positively related to reproductive capacity, resistance to predators, and competiveness for space (Bayne et al., 1983; Dayton, 1971; Paine, 1974, 1976; Robles et al., 1990). Therefore, investigations of environmental-physiological interactions that potentially modify growth rate in *Mytilus*, such as the positive effects of resource acquisition (e.g. dietary composition and quality, ingestive processes, digestive strategies) (Bayne et al., 1987, 1988; Bracken et al., 2012; Dowd et al., 2013; Navarro and Winter, 1982; Riisgard and Randløv, 1981), as well as the negative effects of environmental stress (e.g. high body temperatures) (Fitzgerald-Dehoog et al., 2012; Jimenez et al., 2016; Schneider, 2008) can elucidate the mechanistic factors that potentially modify patterns of mussel distribution and abundance over intertidal landscapes.

**Fig. 1.**
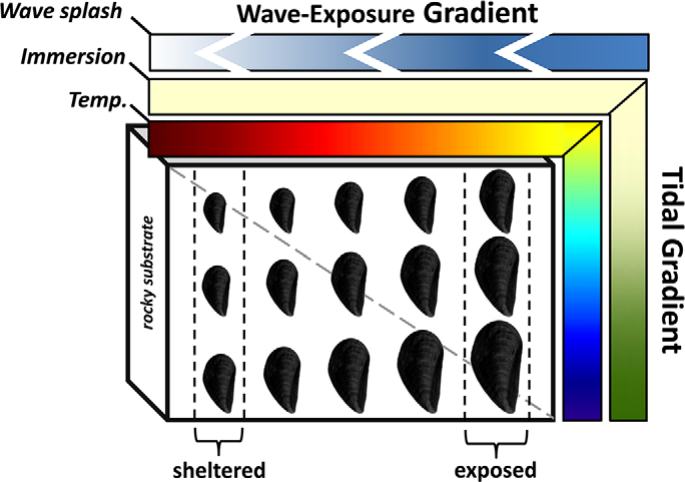
**Schematic showing the effects of environmental and biological gradients on growth in *M. californianus*.** Mussels are attached to a rocky substrate (cubed solid) at three shore levels subjected to a tidal (vertical) and wave-exposure (horizontal) gradient. A wave splash gradient (top color bar) persists from right to left (indicated by direction of chevrons) creating wave-exposed and wave-sheltered segments of shore. Intensity (blue color) attenuates from right to left. An immersion gradient (indicated by green color) persists along the tidal gradient. No gradient (solid color) in immersion exists along the wave-exposure gradient. Temperatures increase continuously (from blue to red) up-shore and towards the sheltered. Similarly, growth rates and body sizes (indicated by size of mussel in the cartoon) vary along the tidal gradient and the wave-exposure gradient. The tidal gradient and the wave exposure gradient are not independent because waves splash water up-shore as well as along-shore (gray diagonal line across the rocky solid).

During low tide the intertidal zone is exposed to solar radiation and the resulting heat influx threatens cellular and organ function in immobile organisms (Braby and Somero, 2006; Gracey et al., 2008; Han et al., 2013; Tomanek and Zuzow, 2010). Because mean tide level varies daily, mean temperatures are lower in low-shore habitats relative to high-shore areas (i.e. tidal temperature gradient; Fig. 1). Temperature also increases along the wave-exposure gradient due to variation in wave splash from wave-exposed to wave-sheltered segments of shore (i.e. wave-exposure temperature gradient; Fig. 1) (Denny et al., 2011; Dowd et al., 2013; Meager et al., 2011; Mislan et al., 2011). In addition, low-shore microhabitats are submerged for longer time periods than high-shore microhabitats (Dehnel, 1956), allowing mussels more feeding time in low-shore areas (see Dowd et al*.*, 2013). However, variation in functional-submergence time (i.e. enough time for optimal feeding) along the wave-exposure gradient is predicted to be constant at any given shore-height. Indeed mussels attached to rocky substrate in wave-exposed segments are subjected to intermittent wave splash (Fig. 1), however no evidence exists that show mussels feed during these short submergence intervals, which are only seconds in duration.

A consequence of prevailing environmental gradients on mussel physiological-ecology is revealed by attenuating indeterminate growth rates up-shore and along decreasing levels of wave-exposure (Fig. 1) (Connor and Robles, 2015; Suchanek, 1981). A mechanistic explanation for these patterns stems from theoretical concepts of bioenergetics. To this end, mussels will only allocate energy toward somatic growth and reproduction after maintenance costs are met (Widdows and Hawkins, 1989), which theoretically include those for repair of temperature-denatured proteins (Gracey et al., 2008). Indeed the sum total of energy within an organism is set by net energy of food that is assimilated during feeding and energy reserves. Therefore, the combined effect of tidally controlled feeding period and level of thermal exposure (duration and intensity) on the energy budget likely explains growth patterns along the tidal gradient (see Sokolova et al., 2012). In contrast, feeding period along the wave-exposure gradient is assumed to be invariable because submergence is set by tidal height. Hence, temperature may have a considerable effect on the variation in growth along the wave-exposure gradient (Connor and Robles, 2015; Fitzgerald-Dehoog et al., 2012).

Digestive enzyme activities (DEA), feeding rates (intake), and assimilation efficiencies (AE) are fundamental parameters used to assess resource acquisition and processing in animals (i.e. net energy gain) (Karasov and Douglas, 2013). Variables of digestive capacity are interrelated as follows:
(1)
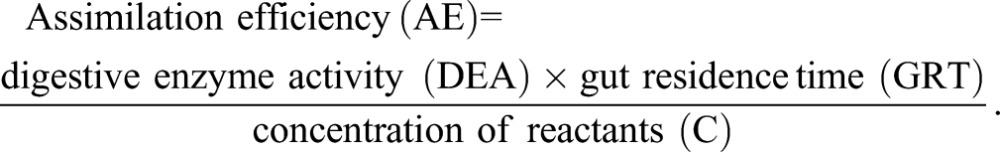


Three of these parameters – digestive enzyme activity (DEA), gut residence time (GRT), and concentrations of reactants (C) – will be affected by intake. Intake affects GRT and C in animals, with high intake meaning smaller GRT and greater C, and thus lower AE, whereas low intake means larger GRT and lower C, meaning greater AE (Karasov and Douglas, 2013). This trade-off between intake and AE is best viewed within the framework of a rate versus yield continuum. For instance, a rate-maximization strategy is characterized by high intake and higher DEA, rapid movement of food through the gut, and relatively low assimilation efficiencies, whereas a yield-maximization strategy is characterized by lowered intake and DEA, slower transit of food through the gut, and higher assimilation efficiencies (German et al., 2015; Karasov and Douglas, 2013). Both strategies are utilized to maximize net energy from available food in the prevailing environment; yield maximization can be used when food is less abundant and rate maximization when food is abundant. Yield maximization often leads to digestion of less tractable components of ingesta (e.g. fiber, like cellulose), whereas rate maximization favors the digestion of soluble components (German et al., 2015). The rate-yield optimization strategy has been shown empirically in *Mytilus chilensiens* and *M. edulis* under varying food concentrations in laboratory-controlled submerged conditions (Navarro and Winter, 1982; Thompson and Bayne, 1972; Widdows, 1978) and modeled computationally in that context (Willows, 1992). Because shore height, the determinant of feeding time, ultimately sets the total amount of food consumed in sessile mussels, it is probable that mussels in nature are rate-maximizers in low-shore habitats, and yield-maximizers in high-shore habitats (Fig. 1).

In this study, we asked whether *M. californianus* adopts rate or yield-maximizing strategies under different circumstances by examining the nutritional physiology of these mussels under laboratory and field conditions. In the laboratory, we asked whether *M. californianus* has a flexible digestive enzyme utilization design and changes its digestive physiology among rate- or yield-maximizing strategies when exposed to variable food concentrations. In the field we asked how rate versus yield strategies are possibly related to microhabitats along gradients of food availability and thermal stress by using digestive enzyme activities as markers of digestive strategy (German et al., 2015; Jhaveri et al., 2015).

We predicted that laboratory mussels exposed to varying food concentrations would display a positive acclimatory response of digestive enzyme activity to increasing food levels, thus fitting a rate-versus-yield continuum. Furthermore, we predicted that digestive enzyme activities in high-shore mussels would be overall lower than activities in low-shore, wave-exposed mussels due to variation in submergence times along the tidal gradient. Although there may be no differences in functional submergence and feeding along the within-shore wave-exposure gradient, we hypothesized that the increased temperature-related stress-costs experienced by the high-shore-wave-sheltered mussels would lead these animals to take more of a yield-maximizing strategy towards digestion to access as much as possible from their food, and the fibrous portions in particular.

## RESULTS

### Physiological performance in laboratory-acclimated mussels

Dry tissue weights increased with the greater availability of suspended food (*P*=0.05), whereas respiration rates for the high-food treatment were marginally higher (*P*=0.06) than the other two food treatments (Table 1). The variance between treatments was not homogeneous. Therefore, the data were log-transformed. An outlier was detected in the normalized low-food treatment data and removed. Clearance rates were marginally significantly higher in the medium food treatment than the other treatments (Table 1; *P*=0.06). Pseudofeces production rates were highest in the high food treatment (*P*=0.03) and lowest in the medium food treatment; the low food treatment wasn't different from either of the other treatments (Table 1). Ingestion rates varied significantly between experimental treatments and were attenuated in the low-food treatment (*P*=0.03). Assimilation efficiencies were significantly higher (*P*<0.001) in the low-food treatment than in the treatments with greater food availability, which didn't differ from one another (Table 1).

**Table 1.**
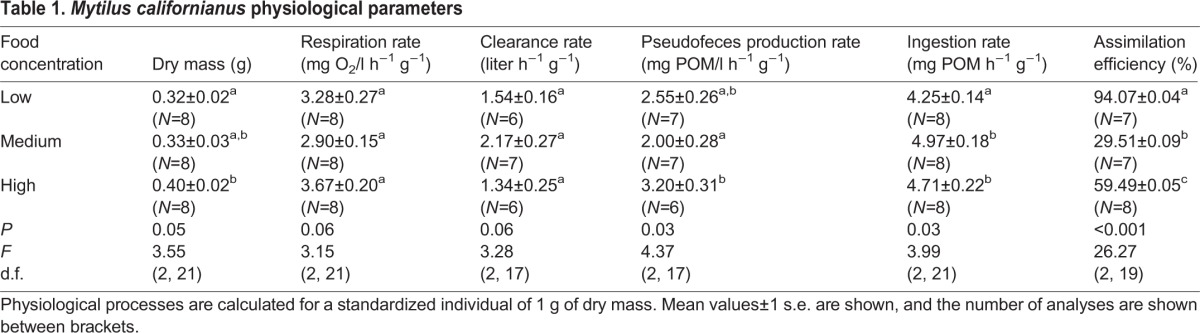
***Mytilus californianus* physiological parameters**

### Digestive enzyme activities in laboratory-acclimated mussels

**Fig. 2.**
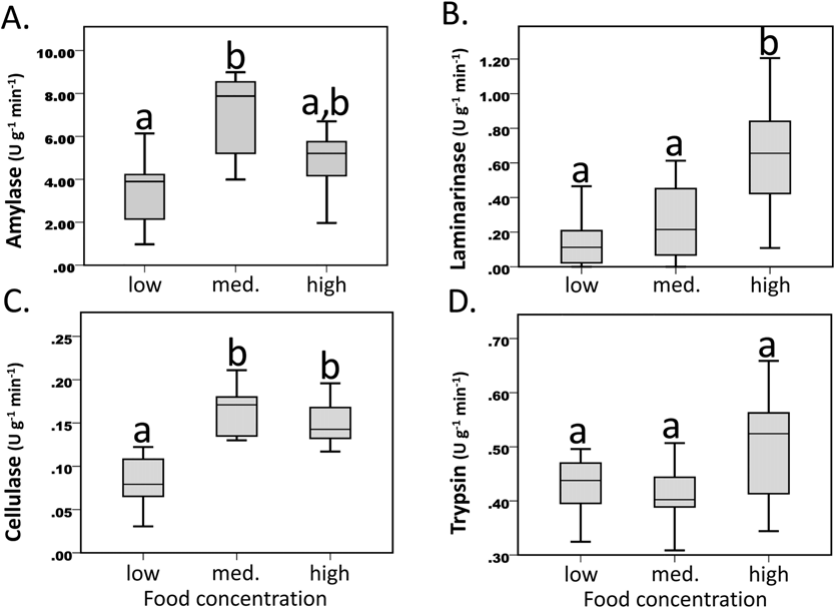
**Laboratory digestive enzyme activities.** Digestive enzyme activity of (A) amylase, (B) laminarinase, (C) cellulase, and (D) trypsin, under laboratory treatments (low, medium and high). Box plots reveal the lower and upper quartiles (box), and the median (line within the box). The adjacent values mark the smallest and largest values not declared as outliers. A one-way ANOVA followed by Tukey's test was used to identify significant differences in mean digestive enzyme activities between treatment groups. The letters indicate the association between treatment groups.

Amylase activity differed significantly (*P*=0.01) between the low and medium treatments while activity in the high treatment was intermediate – not varying from the other two treatments (Fig. 2A; Table S1). Alternatively, laminarinase showed a linear-type response (*P*=0.01) with increasing food supply (Fig. 2B; Table S1). Cellulase activity also showed significant differences (*P*<0.001) between treatments, with the low-food treatment showing the lowest values while the medium and high-food treatments were not significantly different (Fig. 2C; Table S1). Differences between feeding treatments were not detected for trypsin (Fig. 2D).

### Correlation between ingestion rates and digestive enzyme activities

Amylase and cellulase activities were positively correlated with ingestion rate (*P*=0.002 and *P*=0.001, respectively), while no correlation was observed for laminarinase and trypsin (Table S2).

### Field environmental measurements

Field measurements of particulate organic carbon (Fig. S2) and relative chlorophyll *a* (Fig. S3) revealed no variation (*P*=0.12 and *P*=0.21, respectively) between water sampled on a single day from wave-exposed and sheltered regions of the Crystal Cove State Park field site.

**Fig. 3.**
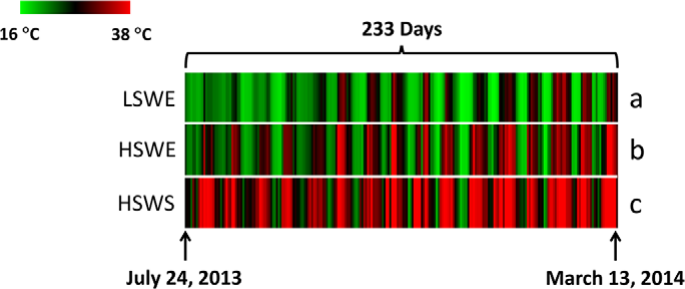
**Temperature conditions at** **Crystal Cove State Park.** Heat-map showing maximum habitat temperatures over a 233-day interval beginning July 24, 2013 at three microhabitats including low-shore-wave-exposed (LSWE); high-shore-wave-exposed (HSWE); and high-shore-sheltered (HSWS). A one-way ANOVA followed by Tukey's test identified significant (*P*<0.05) differences in maximum habitat temperatures between microhabitats, as indicated by letters (see the Results section for s.e.m. values).

**Fig. 4.**
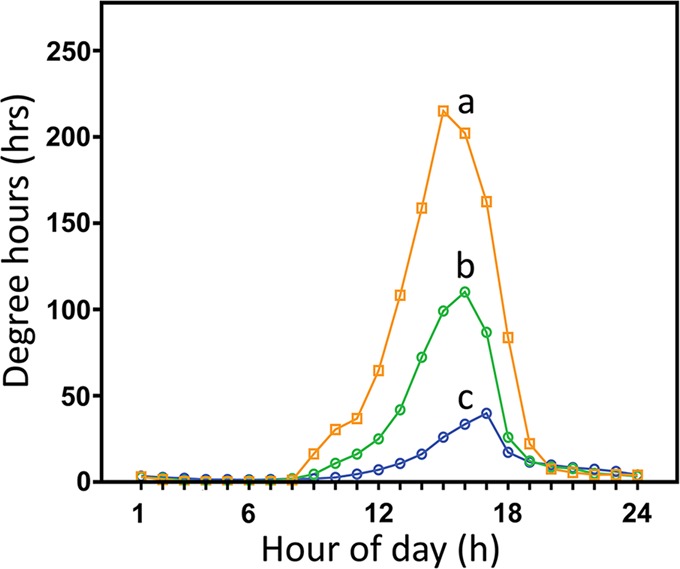
**Degree-hours values at Crystal Cove State Park.** Degree-hours over a 30-day interval beginning September 1, 2013 at three microhabitats including low-shore-wave-exposed, diamonds; high-shore-wave-exposed, circles; and high-shore-sheltered, squares. ANOVA was significant, *P*<0.05, for effects of micro-site on mean degree-hours above 19°C. Letters represent homogenous sites.

The variation in maximum habitat temperatures and degree-hours (above 19°C) between experimental plots was pronounced. The mean maximum temperature differed significantly (*P*<0.001) between microhabitats from July 24, 2013 and March 13, 2014 and were 22.38±0.30 (mean±1 s.e.), 26.16±0.38, and 31.04±0.38°C at the low-shore-wave-exposed, high-shore-wave-exposed and high-shore-wave-sheltered microhabitats, respectively (Fig. 3). In September of 2013 the mean maximum temperatures and degree-hours (above 19°C) increased with increasing tidal height and towards the shore. Maximum temperatures were 20.41±0.42, 24.02±0.81 and 28.71±1.05°C (*P*<0.001) (Fig. S4) and degree hours were 9.21±2.13, 22.58±6.89, and 47.12±14.21 h (*P*=0.02) (Fig. 4) at the low-shore-wave-exposed, high-shore-wave-exposed and high-shore-wave-sheltered microhabitats, respectively. Heat shock protein induction temperature is ∼25°C in *M. californianus* (Buckley et al., 2001) and this temperature was met or breached one, ten, and twenty-one times at the low-shore-wave-exposed, high-shore-wave-exposed and high-shore-wave-sheltered microhabitats, respectively (Fig. S4).

### Field biochemical measurements

**Fig. 5.**
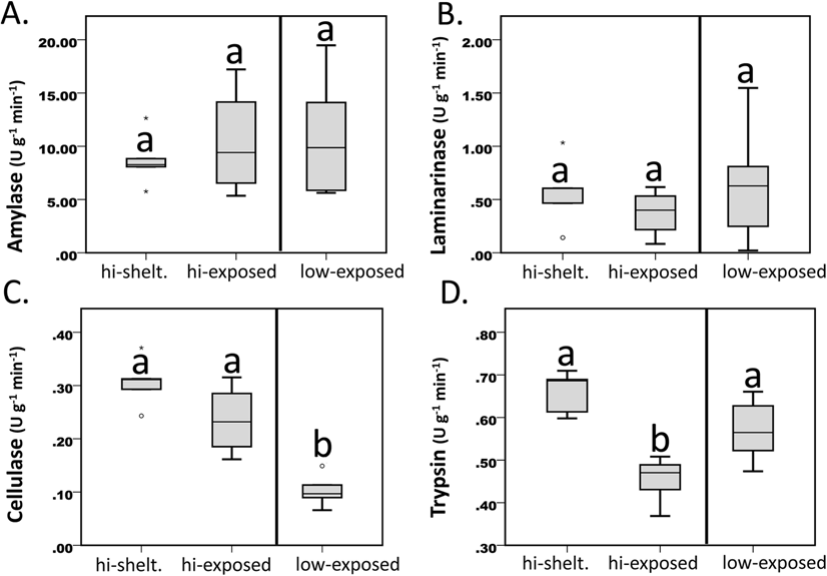
**Field digestive enzyme activities.** Digestive enzyme activity of (A) amylase, (B) laminarinase, (C) cellulase, and (D) trypsin in mussels sampled (*N=*5) from experimental plots. Box plots reveal the lower and upper quartiles (box), and the median (line within the box). The adjacent values mark the smallest and largest values not declared as outliers. A one-way ANOVA followed by Tukey's test was used to identify significant differences (*P*<0.05) in mean digestive enzyme activities between treatment groups, indicated by letters.

Field digestive enzyme analyses showed no variation between microhabitats for amylase and laminarinase (Fig. 5A,B; Table S3). However, cellulase activity was significantly higher (*P*<0.001) in the high-shore mussels (wave-sheltered and wave-exposed) than in the low-shore-wave-exposed mussels (Fig. 5C; Table S3). Trypsin activity was significantly higher (*P*<0.001) in the high-shore-wave-sheltered mussels than the high-shore-wave-exposed mussels, but the activities of this protease were not different between mussels from the two extremes (high-shore-wave-sheltered versus low-shore-wave-exposed) (Fig. 5D; Table S3). As expected, field enzyme activities were higher than those from laboratory conditions, possibly due to higher quality and diversity of food in natural conditions.

**Fig. 6.**
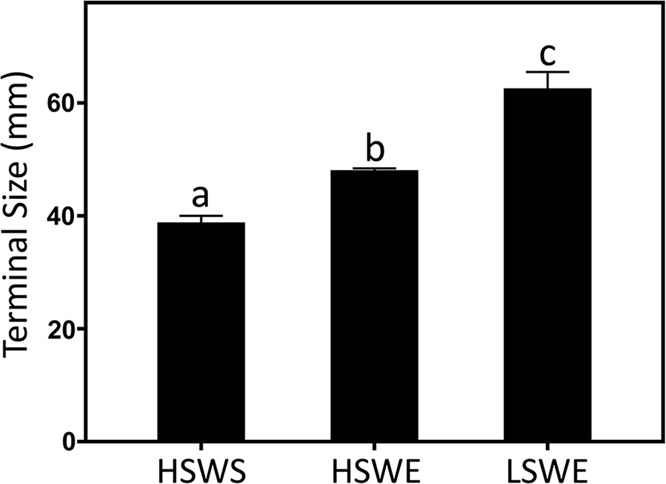
**Mussel size of Crystal Cove State Park samples.** Mean maximum mussel sizes from high-shore-wave-sheltered (HSWS), high-shore-wave-exposed (HSWE) and low-shore-wave-exposed (LSWE) microhabitats. Bars indicate 1 s.e. around the means. A one-way ANOVA followed by Tukey's test identified significant (*P*<0.05) differences in maximum sizes between groups, as indicated by letters.

Maximum sizes decreased up-shore and toward sheltered microhabitats (*P*<0.001) and differences were particularly pronounced between the low-shore-wave-exposed, high-shore-wave-exposed and high-shore-wave-sheltered mussels (Fig. 6). The mean maximum size of mussels from the low-shore, wave-exposed microhabitat was 61% greater than the mean size at the high-shore-wave-sheltered microhabitat.

The mussel explant experiment revealed spatial variation in stress. Percent mortality was 0%, 10% and 80%, in the low-shore-wave-exposed, high-shore-wave-exposed and high-shore-wave-sheltered microhabitats, respectively.

**Fig. 7.**
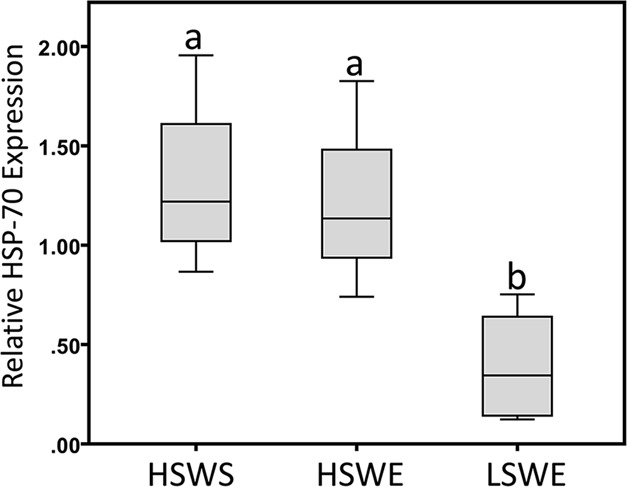
**Expression of *HSP-70* in Crystal Cove State Park samples.** Heat-shock-70 gene expression from mussels (*N=*4) samples from high-shore-wave-sheltered (HSWS), high-shore-wave-exposed (HSWE) and low-shore-wave-exposed (LSWE) microhabitats. Bars indicate 1 s.e. around the means. A one-way ANOVA followed by Tukey's test identified significant (*P*<0.05) differences in maximum sizes between groups, as indicated by letters.

Transcript abundance analysis of *HSP-70* revealed significant differences between microhabitats (*P*=0.02) (Fig. 7). Messenger RNA levels trended with maximum temperatures, degree-hours, and growth rates, with levels increasing up-shore.

## DISCUSSION

Overall our findings suggest that *M. californianus* optimizes feeding and digestion by changing resource acquisition strategies when exposed to different environmental conditions, and utilizes rate- or yield-maximization strategies under different environmental conditions that lead to variable assimilation efficiencies of resources. In the laboratory, assimilation efficiencies were elevated when mussels were exposed to low-food concentrations relative to when mussels were exposed to high-food concentrations. These data suggest that digestion of polymers and absorption of nutrients through the intestinal wall was high when food concentration was low, implying that residence times of food in the gut were longer when ingestion rates were low.

At some upper limit of digesta in the gut, digestive capacity becomes limited and efficiency is compromised (Riisgård et al., 2011). The amount of food that saturates the gastrointestinal (GI) tract varies between food types, which vary in size and nutrient composition. For instance Pascoe et al. (2009), reported that clearance rates were reduced as a result of saturation after feeding for >2 h at ≥30,000 *Isochrysis* *galbana* cells ml^−1^ in *M. edulis*, while Riisgård et al. (2013) found reduced feeding rates at ∼6000-7000 *Rhodomonas salina* cells ml^−1^. In the present study, mussels had slightly reduced clearance rates and elevated pseudofeces production rates when food concentrations were above ∼26,000 cells ml^−1^. However, the experimental mussels were subjected to a mixed diet as opposed to a single algal taxon which was used in prior investigations, thereby complicating direct comparisons between studies.

Our results agree with a laboratory study of *M. edulis* (Thompson and Bayne, 1972), which also revealed a negative relationship between algal food concentration and assimilation efficiency across a range of diet concentrations from 1000 to 25,000 cells ml^−1^ (Thompson and Bayne, 1972). In agreement with their findings and to the logic of nutrient induced rate-yield compensatory adjustments, Bayne et al. (1984, 1988) showed a positive relationship between gut residence time and assimilation efficiency in several mytilid species subjected to an upper limit of algal cells of 12,000 cells ml^−1^. Alternatively, Albentosa et al. (2012) found a positive relationship between assimilation efficiency and food availability in *M. galloprovincialis* acclimated for six days in the laboratory. However, all of the food concentration treatments used by Albentosa et al. (2012) [0.50-1.80 mg l^−1^ particulate organic matter (POM)] were below the lowest treatment concentration (5.50 mg l^−1^ POM) in the present study, and the short acclimation period (six days) may have only captured an acute response. To this end, the POM range of our study encapsulates the range of POM values along coasts of the eastern Pacific (Díaz et al., 2014; Luna-González et al., 2008; Page and Ricard, 1990).

The positive relationship between food concentration and respiration rate has been shown in several studies across a range of bivalve taxa (Albentosa et al., 2012; Griffiths and King, 1979; Hawkins et al., 1986; MacDonald et al., 1998; Thompson and Bayne, 1974) including *M. californianus* (Dahlhoff et al., 2002), and is indicative of variation in feeding costs (e.g. mechanical activity of the gill pump) and specific dynamic action (SDA – energy expended from the ingestion, digestion, absorption; Secor, 2009). A study by Bayne and Scullard (1977) found that 24% and 4% of the energy available in an ingested ration was attributed to mechanical costs of feeding and SDA, respectively. In the present study, mussels that were acclimated to high food concentrations and exhibited elevated tissue growth, displayed marginally higher respiration costs which comes as a consequence of greater levels of food within the gut and pronounced size-related digestive and maintenance costs. In the context of environmental adaptation, Albentosa et al. (2012) interpreted the close relationship between respiration and feeding costs in *M. galloprovincialis* as a mechanism to minimize the inefficient use of endogenous resources under conditions of limited resources.

Unique to this study is the first observation of a concerted positive response of mass-specific enzyme activities of amylase, laminarinase and cellulase in the digestive gland of *M. californianus* to increased food quantity, under relatively long-term (four weeks) treatment conditions. In general, enzyme activities increased from low to medium food-level treatments suggesting that the digestive gland acclimates to maximize nutrient acquisition between these feeding levels – in agreement with a digestive system with a flexible design. Interestingly, under high food levels, the carbohydases, amylase and cellulose, were down-regulated while laminarinase activity continued to elevate between medium and high food levels. The down-regulation of enzyme activity at high ingestion rates is in agreement with nutrient-balancing principles, which assumes homeostatic functionality of the GI system under conditions of variable diet (Clissold et al., 2010). For example, once the need for a particular nutrient within a given diet is met by dietary intake, an organism will subsequently down-regulate the complementary digestive machinery (i.e. digestive enzyme activity) to further acquire it.

Previous studies show that bivalves also fulfill energy requirements via uptake of dissolved organic material (DOM) across various tissues (Ferguson, 1982; Manahan et al., 1983; Uchida et al., 2010). For example Gorham (1988) showed that DOM under natural conditions can make up 13% and 10% of energy and nitrogen requirements respectively in *M. edulis*. It is likely that the level of DOM in our tanks positively scaled with food concentration and could have contributed to variable digestive enzyme activity or putative nutrient balancing processes. However, the effect of variable DOM on digestive enzyme activity has not been shown in bivalves. In this context, a recent review of the exploitation of DOM by marine invertebrates states that the ecological benefits of DOM for these organisms remains largely unknown (Wendt and Johnson, 2006). Simultaneous studies of DOM uptake and digestive enzyme activity within the same individual are needed to fully comprehend these complexities in lab-based experiments and in nature.

In the present study, the preponderance of post-ingestive strategies was possibly a response to the nutrient complexity of a multi-algal diet that included four cell types that differed in their carbon:protein ratio. The mixed diet consisted of a combination of green algae, golden/brown microalgae and diatoms (straminopiles); the diatoms composed 20% (w/w) of the feed and they are the only component that contained a form of laminarin (i.e. chrysolaminarin) (Sakamoto and Toyohara, 2009). Diatoms contain much higher cellular levels of 20:5 (n-3) and 22:6 (n-3) polyunsaturated fatty acids (PUFAs) than any other microalgae. PUFAs have been identified as essential nutrients within the diets of bivalves (Brown et al., 1997). Therefore, it is possible that mussels in the high-feed treatment continued to select for the small proportion of diatoms in the feed, post-ingestively, even while attenuating their digestion of the starchy, less-essential microalgal species. Similarly, Albentosa et al. (2012) observed in *M. galloprovincialis*, a negative response in protease activity and a simultaneous positive response in amylase, in the face of rising concentration levels of a single species algal diet. Post-ingestive compensations are potentially necessary strategies that help elevate and maintain the sum total energy budget, in the face of decreased ingestion rates of mussels high on the shore (Albentosa et al., 2012).

Results from the feeding behavior and digestive enzyme activity laboratory experiments provided context to observations of bioenergetic variation along environmental gradients within the intertidal zone, although because of obvious differences in physical variables, we did not make direct comparisons between laboratory and wild-caught animals. Environmental variables that affect the sum total of the energy budget such as tide-level, temperature and food concentration, change frequently (i.e. minutes to days) within a micro-site and between sites (Denny et al., 2011). However, our long-term (>7 months) temperature data revealed a consistent pattern of higher daily habitat maximum temperatures up-shore and toward sheltered microhabitats. Consistent with Connor and Robles (2015), temperature was negatively correlated with maximum size which further suggests that temperature stress plays a pivotal role in the allocation of energy toward growth. Proxies of food (POC) did not vary between the wave-exposed and sheltered segments of shore at a given shore height, which strongly suggests that any variation in feeding that might exist is the result of endogenous mechanisms such as feeding behavior (e.g. valve gape; particle sorting) or digestive processes (e.g. digestive enzyme activity). However, Dowd et al*.* (2013) revealed that the ATP-generating enzyme citrate synthase in *M. californianus* did not vary between intra-site wave-exposed and sheltered ends of shoreline. Their results may reflect invariable food intake along the wave-exposure gradient.

An assumption of this discussion is that enzyme activity increases with food availability, which decreases along the tidal gradient within the intertidal zone. Remarkably, we observed elevated activity levels of cellulase and trypsin in high-shore-wave-sheltered microhabitats as opposed to the predicted positive relationship between digestive enzyme activity and levels of submergence (i.e. food availability). Cellulose, a component of the cell walls of green algae and found in detritus, contains beta-bonds that are more structurally resilient (Hummel et al., 2006) than starches. However, rather than matching digestive investment with food availability, which was revealed in our laboratory experiment, it appears that mussels with less opportunity to feed (due to aerial exposure) and experiencing greater thermal stress compensate for lower energy scope by overproducing cellulase, which is suggestive of ‘scavenging’ behavior and consistent with a yield-maximizing strategy.

Similarly, Moal et al*.* (1989) found that amylase activity in the intertidal oyster *Crassostria gigas* was greater in individuals explanted high on the shore than those installed mid and low-shore, while adenylate energy charge remained constant. Alternatively, Labarta et al. (2002) revealed higher digestive enzyme activities in mussels transplanted to submerged conditions than individuals acclimated to a tidal flat. However, unlike Moal et al. (1989), the nutrient conditions between habitats differed greatly. Hence, the effects of submergence time and food concentration on digestive enzyme activity could not be teased apart. Lastly, Elvin and Gonor (1979) showed that *M. californianus*, acclimated to bouts of aerial exposure displayed greater assimilation of labeled algal cells than mussels acclimated to submerged conditions. Therefore, it is a reasonable expectation that mussels high on the shore, which are unable to egest feces during aerial exposure, have longer GRT and use yield-maximization strategies (including cellulose and fiber digestion) in order to sustain net energy to survive (i.e. not to fully match net energy of mussels low-shore) under conditions of limited feeding time. The enrichment of digestive enzyme activity in high-shore mussels may also be reflected in the lack of variation of the other carbohydrase activities between low and high-shore microhabitats. However, the wider variation in amylase and laminarinase (which digest more-soluble molecules) activity displayed by low-shore mussels suggests a greater opportunity to feed on resources in this microhabitat versus high-shore segments of shore.

We infer that the particular investment in cellulase enhances survival by bolstering ATP resources that are critical for maintaining energy balance in environments to which the energetic buffer between life and death is narrowed. Cellulose can be considered low-quality food that is abundant in components of near-shore detritus. *Mytilus californianus* prefers higher quality food (Bracken et al., 2012), which may be reflected in variable regulation in digestive enzyme activity. That is, high-shore mussels up-regulate cellulase activity in the face of limited feeding time and low-shore mussels down-regulate cellulase activity because of more opportunity to select high-quality food. In support of this inference, Charles and Newell (1997) found that absorption efficiency of ^14^C-labeled lignocellulosic detritus was higher in *Geukensia demissa* mussels subjected to emergence than those acclimated to constant submergence, similar to comparing our high-shore-wave-sheltered mussels to the low-shore-wave-exposed mussels. Whether the cellulase we measured is endogenous (i.e. synthesized and produced by the mussels, which have cellulase genes in their genome; Xu et al., 2001) or exogenous (i.e. produced by microbes residing in the mussel digestive tract or coming in with the food), is unknown, but both can play a role in cellulase activity variation (German and Bittong, 2009; Karasov and Douglas, 2013).

Similarly, trypsin activities varied between the high-shore-wave-sheltered and high-shore-wave-exposed microhabitats. The elevated digestion of proteinaceous substrates by trypsin in the high-shore-wave-sheltered mussels may also allow for greater protein acquisition for enhanced survival in the face of reduced feeding times. Moreover, pronounced amino-acid absorption may be a necessary function of mussel digestive machinery in order to effectively deal with constant re-synthesis of proteins lost from irreversible denaturing from thermal perturbations that occur during low-tide. The results from measurements of three indices of stress (number of heat-shock days, percentage mortality of explanted mussels, and induction of *HSP-70*) at spatially separated microhabitats were consistent with the spatial patterns of habitat temperatures across the landscape of the study site – maximum temperatures, habitat degree-hours and stress indices, increased up-shore and toward sheltered microhabitats. Stress in high-shore populations have also been confirmed in prior field-based studies that showed elevated levels of sequestosome mRNA transcripts (proteins involved in the degradation of mRNA), ubiquination (the process of tagging proteins destined for degradation), lipid peroxidation and heat shock proteins (Gracey et al., 2008; Halpin et al., 2004; Hofmann and Somero, 1995; Jimenez et al., 2016; Roberts et al., 1997). Hence, mussels high on the shore and away from cooling effects of wave splash may be particularly vulnerable to future increases in climate temperature as a result of global change.

### Concluding remarks

*Mytilus californianus* beds resemble vital chemical reactors within shoreline environments because they convert suspended organic material into sinking particulate organic material in the form of feces, as well as dissolved organic substances such as ammonium (Bayne et al., 1976). Its contribution to biogeochemical processes and trophic cascades of nearshore regions is proportional to its demography (e.g. distribution, size, weight) (Prins et al., 1997), which in turn is modified by environmentally sensitive physiological processes (e.g. feeding, growth, reproduction). Connor and Robles (2015) revealed consistent patterns of growth, temperature and wave force along the tidal-wave-exposure vector (Fig. 1). A critical finding in that, and the current study, was that mussels high on the shore and furthest from wave splash endure greater average temperatures which likely result in elevated levels of organismal stress and smaller sizes. Thermal stress leads to increased use of ATP in order to reassemble affected proteins (Lindquist and Craig, 1998). However, ATP levels can be restored by the acquisition of energy stored in food (Sarà et al., 2011). Mussels are well adapted to their environments and compensate for fluctuations in bioenergetic stressors through the gut (e.g. digestive enzyme activity) and peripheral cellular adjustments (e.g. heat-shock protection) in order to survive. Previous studies and data reported here, suggest that mussels in sheltered intertidal regions display a yield-maximizing strategy; hence, they exhibit greater assimilation efficiencies, scavenge more recalcitrant substrates such as cellulose, and up-regulate enzymes that digest such substances (Charles and Newell, 1997; Elvin and Gonor, 1979). It is important to note that while there is a positive correlation between ingestion rate and some enzymes in the lab, the enzyme activity we observed in this study is likely tied to mechanisms resulting from long-term acclimation in the lab and acclimatization in the field. Finally, along the wave-exposure gradient, acclimatized-compensatory responses in mussels at some critical distance away from the splash zone are overcome by the energy demands imposed by (chronic) thermal stress, and the inability to survive at these distances abrogates their horizontal distribution (see Robles and Desharnais, 2002). Long-term landscape physiological-ecology studies will help to resolve these assumptions.

We took a snapshot approach at observing digestive enzyme activity in the field (i.e. a single time point). In this regard, Langton (1977) found no significant variation in amylase activity between periods of emersion and immersion in *M. edulis*, but Moal et al. (2000) showed a small and lagged response of amylase activity following pulses of food. Hence, our digestive enzyme activity measurements likely reflect stored enzymes (as zymogens). An abundance of work is needed to fully resolve these complex interactions. For example, environmental simulations will allow for high-frequency temporal-based sampling to capture exogenous and endogenous rhythms, closer monitoring of physiology (e.g. ingestion rate), comprehensive analyses of digestive physiology (e.g. digestive enzyme activity, GRT), and their interactions. Enzyme activities in the field were higher than those in the laboratory, thereby highlighting possible shortcomings of food supplements, behavioral differences between these habitats and the effects of DOM (Alfaro, 2006). Nonetheless, the current study improves our understanding of the link between digestive flexibility, bioenergetics, and ecology in *M. californianus*; a rarely explored set of integrated processes in the purview of intertidal research.

## MATERIALS AND METHODS

### Laboratory acclimations for digestive enzyme activity

A sample of 21 mussels (∼6.5 cm in length) were collected from a single low-shore microhabitat +0.40 m above mean lower low water (MLLW) on a rocky headland within Crystal Cove State Park, Laguna Beach, California (33° 33′ N, 117° 49′ W) (all mussels were collected similarly throughout the study). They were cleaned of epibionts, placed in a 189-liter closed aquarium filled with gravel-filtered seawater and kept at 17°C and salinity of 35 ppt. Mussels were allowed to depurate for three days, and were then transferred to three treatment (76 liter) closed aquaria (seven mussels per tank) and allowed to acclimate for 4 weeks. Treatments, including relative low, medium and high-food level conditions, were prepared by supplementing tanks with Shellfish Diet (Reed Mariculture, Campbell, CA). Shellfish Diet is composed of a per dry weight mixture of *Isochrysis sp.* (golden/brown flagellate) 40.0%, *Pavlova sp* (golden/brown flagellate) 15%, *Thalossiosira weissflogii* (diatom) 20.0%, and *Tetraselmis sp.* (green flagellate) 25.0%, which collectively had a nutritional composition of 52.0% protein, 16.1% lipid and 22.0% carbohydrate. The food supplement treatments were based on 0.2%, 1% and 5% of live tissue weight which was estimated with the average length of the cohort using the equation y=0.5082e^0.0366*L*^; where *L*=posterior-anterior length in mm [determined from data in Suchanek (1981)]. The 5% treatment is optimal for growth, according to manufacturer's instructions. A computer-controlled peristaltic pump was used to add the food to the aquaria daily. Suspension of food particles was maintained by water pumps placed at opposite ends of each aquarium. A submerged foam filter pump was activated for 3 h daily to remove uneaten food and suspended feces. Half of the water in each tank was changed weekly. Following acclimation, mussels (*N=*7 per treatment) were sacrificed and the digestive gland was removed and immediately frozen on dry ice and stored at −80°C.

### Digestive enzyme activity measurements

#### Carbohydrase assays

Digestive glands were weighed, then homogenized in 25 mmol l^−l^ maleate buffer, pH 6.5 [the pH of the digestive gland in *M. edulis* in air and water; see Langton (1977)] with a Polytron PT 10-35 homogenizer (Brinkman Instruments, Westbury, NY) at 3000 rpm for 3×30 s, and centrifuged at 9400×***g*** for 2 min at 4°C. Following centrifugation, the supernatants were collected and stored in small aliquots (100-200 µl) at −80°C until just before use in spectrophotometric assays of activities of digestive enzymes. Assays measuring activities of amylase, laminarinase, and cellulase were carried out with the Somoygi-Nelson method, as described by German and Bittong (2009). 50 µl of substrate was combined with 45 µl of buffer (25 mol maleate and 1 mmol CaCl_2_ at pH 6.5) and 5 µl (amylase and cellulase) or 10 µl (laminarinase) of homogenate in 1.5 ml centrifuge tubes. The incubation phases of all assays were carried out at 17°C for 30 min for amylase and laminarinase, while 2 h under constant shaking was used for cellulase. 1% starch, 1% laminarin and 0.5% carboxymethyl cellulose and were used as the substrate concentrations for amylase, laminarinase, and cellulase activity measurements, respectively. Following incubation, Somoygi-Nelson reagent A was added. Reagent B was added after 10 min of boiling with the Somoygi-Nelson reagent A and cooling on ice. Following centrifugation, the absorbance of the supernatant was recorded by a spectrophotometer (BioTek Synergy H1, Winooski, VT) at a wavelength of 650 nm. Net absorbances were calculated by the difference in absorbance between homogenate and substrate controls and the reaction mixture. Activity was determined with a glucose standard curve. Mass-specific enzyme activities are expressed in U (1 µmol l^−l^ reducing sugar liberated per minute) per gram wet weight digestive gland tissue.

#### Trypsin assay

The trypsin assay was carried out in 25 mmol l^−1^ maleate at pH 6.5. The substrate was produced by dissolving 0.01 g of N-alpha-benzoyl-l-arginine-p-nitroanilide (BAPNA) with heat. 90 µl of cooled BAPNA solution was combined with 10 µl of homogenate or buffer to form the reaction mixture and substrate blanks, respectively. Activities were calculated by the difference in absorbance between the reaction mixture and substrate blanks at 410 nm. Trypsin activity was determined with a p-nitroaniline standard curve, and expressed in U (1 µmol p-nitroaniline liberated per minute) per gram wet weight of digestive gland tissue.

### Physiological measurements

Physiological measurements including respiration rate, clearance rate, ingestion rate, pseudofeces (i.e. regurgitation) production rate, and assimilation efficiency were recorded for 24 mussels (eight mussels per treatment) following acclimation, as previously described. Collection and treatment protocols were similar to that of the digestive enzyme experiment.

Respiration rate (RR) during feeding was carried out in a respirometer; a 500 ml chamber outfitted with an optical oxygen sensor (Ocean Optics, Dunedin, FL). The sensor was calibrated with fully aerated (100% O_2_) and anoxic (0.00% O_2_) seawater. Each mussel was placed in the chamber filled with seawater under constant stirring. Shellfish Diet was introduced by inserting a needle, attached to a syringe, into the chamber. Each individual was allowed to acclimate for 1 h to allow valves to open, before O_2_ consumption was recorded. Respiration rate was measured as the difference in %O_2_ before and after the recording period. %O_2_ was converted to mg l^−1^ (at 35 ppt and 17°C) by the conversion 1% O_2_=0.078 mg l^−1^. Respiration rate expressed was calculated for each mussel with the following equation: RR=(C_0_ – C_t_)/t where C_0_ and C_t_ are the initial and final O_2_ concentrations and t is time.

Clearance rate is defined as the volume of water cleared of particles, and used in place of intake rate in bivalve literature. Clearance rate was measured by placing mussels in 1 liter vessels. Mussels were allowed to acclimate for 1 h under constant stirring, then 1 ml of water was sampled from the beaker and every 15 min thereafter for 45 min and cell concentration (cells ml^−1^) measured with a Coulter Counter (Beckman Coulter, Indianapolis, IN). Clearance rate (CR), expressed as liter h^–1^, was calculated with the equation: CR=(V/ t×n)×(ln(C_0_/C_t_)) where V is volume of the vessel, t is time, n is the number of mussels, and C_0_ and C_t_ are the initial and final concentrations of particles in the water (Riisgård et al., 2011). Pseudofeces production rate (PR) (i.e. pseudofeces as a function of pumping rate) was calculated as mg organic pseudofeces/clearance rate, and expressed as mg POM/l h^–1^. A total of seven, seven, and six mussels displayed active feeding (open valves) in the low-, medium- and high-food treatment, respectively and these particular individuals were analyzed for clearance rates.

Assimilation efficiency measurements were conducted in 1 liter glass vessels under constant stirring. Mussels were starved in organic-free saltwater (Instant Ocean, Spectrum Brands, Blacksburg, VA) for 24 h prior to treatment. Following starvation, mussels were allowed to feed for 2 h under each treatment in natural seawater (identical concentrations as clearance-rate treatments). After the feeding period, pseudofeces were removed with a pipette, followed by the placement of each mussel in separate vessels containing organic-free saltwater. A pipette was used to remove feces 24 h later (see Wang et al., 2015).

Pseudofeces and feces were placed on pre-combusted and pre-weighed Whatman GF/C filters (37 mm) and the organic (ash-free dry weight) and inorganic constituents (ash dry weight) were measured. Filters were dried at 65°C overnight followed by 105°C for one hour and weighed. Filters were then combusted at 550°C for three hours and re-weighed. The organic material of Shellfish Diet and seawater were determined similarly.

The POM levels of the sea-water-Shellfish Diet mixture were ∼5.48, 8.03, and 15.33 mg l^−1^ between low, medium and high food levels, respectively. The organic to inorganic ratios were 0.69, 0.84 and 1.07 from low to high food levels. To account for metabolic fecal loss, feces organic values were reduced by 15% of ingested food across all treatments, in accord with a study of *M. edulis* (Hawkins et al., 1990). Total mass of the organic material ingested, hence the ingestion rate (IR) expressed as mg POM h^–1^, was estimated by the association of inorganic material in the feces (the indigestible portion of ingested food) with the organic:inorganic ratio of food: IR=inorganic dry weight_feces_×organic dry weight_food_/inorganic dry weight_food_.

Assimilation efficiency was calculated with the following equation: %AE=100×[(I-F)/F] (where I is ingested organic material, and F is feces).

All physiological performance measurements were standardized to 1 g dry tissue mass using the equation Ys=(Ws/W)^0.67^×Ye. Ys is the standardized variable, Ws is the standard mass (1 g), W is the measured dry mass of the individual, and Ye is the physiological measurement. The allometric exponent (0.67) was determined for mussels by Bayne and Newell (1983).

### Field site description

A discontinuous (i.e. broken in two parts) headland at Crystal Cove State Park, CA (33° 33′50″N; 117° 49′44″W) (Fig. S1), was used as the location to measure variation in digestive enzyme activity and heat stress in mussels between three microhabitats: low-shore-wave-exposed, +0.40 m above mean lower low water (MLLW); high-shore-wave-exposed, +0.90 m above MLLW; and high-shore-wave-sheltered, also +0.90 above MLLW, but 16 m shoreward from the high-shore-wave-exposed site (Fig. S1). Thus, microhabitats were observed at the extreme ends of the tidal and wave-exposure gradients. The shore levels were established with a Topcon (Itabashi-ku, Tokyo) totaling station (Connor and Robles, 2015). The face of waves traveling toward the shore were approximately perpendicular (90°) to the axis of the headland, which contained the mussel bed on its south face (∼45° incline). Waves crashed at the tip of the headland causing wave energy to dissipate shoreward.

Microhabitat temperature was estimated with a single Tidbit temperature logger (Onset computers, Bourne, MA) embedded in a resin disc (diameter=7.62 cm and height=1.91 cm) placed at each microhabitat on July 24, 2013 and habitat temperatures recorded every 10 min for 233 days. From these recordings we determined habitat maximum temperature and degree-hours. Degree-hours was calculated as the total number of hours spent above 19°C – the lower limit of aerial temperature between microhabitats.

Variation in suspended nutrients in water between wave-exposed and sheltered ends of the shore was estimated by measuring particulate organic carbon (planktonic biomass and detrital approximate) and relative chlorophyll *a* in water collected from each microhabitat. On April 4, 2014, 1 liter bottles were filled with seawater collected from the wave-exposed end and sheltered end of the horizontal transect (*N*=6 per micro-site) during mid-tide. The total time of collection was 15 min. 200 ml per assay replicates were passed through a Whatman GF/F filter (25 mm) (0.70 m filter pore size) under low pressure (5 psi) and the filtered particles were analyzed. The samples did not contain large (visible to the eye) fragments of organic material. Particulate carbon (PC) was dried at 80°C for 48 h before pelletizing samples and analyzing them with a Flash EA 1112 series NC elemental analyzer (Thermo Scientific, Waltham, MA) (Garcia et al., 2015). Chlorophyll *a* was extracted in 90% acetone at −20°C for 24 h (Knap et al., 1996) before reading the absorbance at 430 nm with a Genesys 10vis spectrophotometer (Thermo Scientific).

### Field biochemical measurements

We measured digestive enzyme activities, to assess variation in nutrient acquisition in the field. Five mussels (4-6 cm) were removed from each microhabitat during low-tide on the morning of July 26, 2013. The digestive glands were removed less than 30 min after mussels were removed from the shore, frozen on dry ice and stored at −80°C.

To approximate organismal stress in each microhabitat, we measured the mortality of mussels explanted to the established microhabitats (low-shore-wave-exposed; high-shore-wave-exposed; and high-shore-wave-sheltered). Thirty mussels were collected from Crystal Cove State Park and allowed to acclimate in the holding tank at UC Irvine for 2 weeks and fed a diet consistent with medium concentration conditions (see Laboratory acclimations for digestive enzyme activity). After the acclimation period mussels were explanted to the three microhabitats (10 mussels each) by securing them under a square sheet of Vexar mesh that was bolted at its corners to the rock surface. The mussels were allowed to acclimatize for 4 weeks and percentage mortality was recorded following the acclimatization period.

Patterns of thermal stress between microhabitats were evaluated by measuring the mRNA level of *HSP-70* on a ‘hot’ day (habitat temperatures were above the heat-shock induction temperature of 25°C) with RT-qPCR techniques. On October 7, 2014 and during hot Santa Ana conditions, mussels (4-6 cm) were collected from each sampling micro-habitat then kept in cool water (17°C) for 1 h. After incubation, gill pieces were removed and frozen on dry ice. The maximum air temperature in Laguna Beach, California was 32°C, while maximum low-tide habitat temperatures prior to sampling were 25.5°C, 29.5°C and 30°C at the low-shore-wave-exposed, high-shore-wave-exposed, and high-shore-wave-sheltered micro-habitats, respectively. Total RNA was extracted with Trizol (Invitrogen) according to manufacturer's instructions and 1 µg of each RNA sample was reverse-transcribed with (Promega) in a 20 µl reaction. 1 µl of the resulting cDNA was used in a 1× SYBR Green mix (BioRad) and amplified with a thermal cycler. Primer sequences for target *HSP-70* (GenBank Accession # ES735872.1) were 5′-3′ TATGGCAGGAAAAGGTCCAC and GCGACTTGATTTTTAGCTGCAT and for reference *Alpha-tubulin* (GenBank Accession # ES735904.1) were 5′-3′ TCCAAGACACGGCAAATACA and TTGAAACCAGTTGGACACCA. All primers pairs exhibited an amplification efficiency >95%, and relative expression was measured with the Δ-ΔCt method (Livak and Schmittgen, 2001).

Variation in growth was estimated by measuring the maximum sizes of mussels at each microhabitat by removing the four largest mussels from within a 21×7 cm quadrat frame haphazardly placed onto the bed surface. The longest dimension of the quadrat was parallel to the horizontal-gradient in order to not confound variation in growth along the tidal gradient with growth along the wave-exposure gradient.

### Statistics

Levine's tests were performed to assess homogeneity of variance between experimental treatments. In cases to which unequal variances were identified, the data were log transformed using the function ln (x+1). The Carling (2000) method which reduces the effect of sample size on boxplot rules, was used when outliers were removed. A one-way ANOVA followed by Tukey's test was used to test the null hypothesis of no difference in the mean values of each performance, enzymatic and field measurement, between independent treatments. A linear regression was used to correlate mean ingestion rate of each treatment and laboratory digestive enzyme activities. A *P*<0.05 was used as the criteria to reject the null hypothesis in each analysis. The mean and standard error (1 s.e.) values were reported for all biological and environmental variables. *HSP-70* expression was calculated using ratios. Ratios do not have normal distributions therefore the Kruskal–Wallis non-parametric test was used to test for differences between treatments. IBM SPSS Statistics™ ver. 20 was used to perform statistical analyses. Outlier detection was determined using R ver. 2.1.0; with package, Rallfun-v27 (Wilcox, 2012).
